# Complementary alternative medicine use among postpartum mothers in a primary care setting: a cross-sectional study in Malaysia

**DOI:** 10.1186/s12906-020-02984-7

**Published:** 2020-06-26

**Authors:** Nik Farah Nik Yusof Fuad, Siew Mooi Ching, Dayangku Hayaty Awg Dzulkarnain, Ai Theng Cheong, Zainul Amiruddin Zakaria

**Affiliations:** 1 Klinik Kesihatan AU2 Keramat, Ministry of Health Malaysia, Taman Sri Keramat, Jalan AU2, Putrajaya, 54200 Selangor Malaysia; 2 Department of Family Medicine, Faculty of Medicine and Health Sciences, Universiti Putra Malaysia, 43400 Serdang, Malaysia; 3 Malaysian Research Institute on Ageing, Universiti Putra Malaysia, Serdang, 43400 Malaysia; 4 Department of Biomedical Science Faculty of Medicine and Health Sciences, Universiti Putra Malaysia, Serdang, 43400 Malaysia

**Keywords:** Complementary alternative medicine, Postpartum mothers, Primary care, Malaysia, Prevalence

## Abstract

**Background:**

Complementary alternative medicine (CAM) is widely used among postpartum mothers to maintain their well-being. This study aims to determine the prevalence and factors associated with CAM use among postpartum mothers in a primary-care clinic in Malaysia.

**Methods:**

This is a cross-sectional study of 725 postpartum mothers, aged 18 and above, attending a primary-care clinic. The systematic sampling method was used to recruit patients through a structured, self-administered questionnaire. Data analysis was conducted using SPSS version 23. Multiple logistic regression was used to identify the predictors of CAM use among postpartum mother**s**.

**Results:**

The prevalence of CAM use among postpartum mothers was 85.5%. Manipulative body therapies, including massage, reflexology, hot stone compression and body wrapping were the most widely used methods of CAM (84.1%) among postpartum mothers, followed by biological-based therapies (33.1%). More than half of the respondents (52.1%) opted to use CAM, as they had observed good results from other CAM users. However, our study showed that 57.1% of mothers who consumed herbal medicine reported neonatal jaundice in their newborn. The median of the expenditure on CAM usage was 250 Malaysian Ringgits, or USD 61.3 per month. According to multiple logistic regression analyses, being Muslim (OR = 5.258, 95% CI: 2.952–9.368), being Malay (OR = 4.414, 95% CI: 1.18–16.56), having a higher educational level (OR = 2.561, 95% CI: 1.587–4.133) and having delivered via spontaneous vaginal delivery (OR: 5.660, 95% CI: 3.454–9.276) had a significantly positive association with CAM use among postpartum mothers.

**Conclusions:**

The prevalence of CAM use was high (8 out of 10) among postpartum mothers. Postpartum mothers who are Malay, Muslim, have a higher educational level and who have had spontaneous vaginal delivery tended to use CAM more. Manipulative body therapies, including massage, reflexology, hot stone compression and body wrapping, were the most widely used forms of CAM, followed by biological-based therapies. More than half of the mothers who consumed herbal medicine reported neonatal jaundice in their newborn. Thus, education to increase awareness regarding the consumption of herbs is urgently required in this country.

## Background

Complementary and alternative medicine (CAM) is defined as a group of diverse medical and healthcare systems that are not conventionally practised or fully integrated into a country’s dominant healthcare system [1]. CAM is commonly classified, according to National Center for Complementary and Alternative Medicine (NCCAM), into the following categories: alternative medical systems, biologically-based therapies, manipulative body therapies, mind–body therapies and energy-healing therapies [2]. In Malaysia, CAM is classified into six groups, including traditional Malay medicine (Malay herbs, cupping and massage), traditional Chinese medicine (acupuncture & moxibustion, Chinese herbs, Chinese cupping and Qi Gong), traditional Indian medicine (Ayurveda, Siddha, Unani, yoga and naturopathy), homeopathy, Islamic medical practices and complementary therapies (mind–body medicine, biological-based therapy, manipulative therapy and energy medicine) [3].

A few studies have found that CAM is frequently used for patients with chronic diseases, such as hypertension and diabetes [4, 5], as well as acute illnesses like dengue and herpes simplex [6–8]. They are also commonly used by postpartum mothers to reduce pain and postpartum blood loss by promoting wound healing and improving uterine recovery, along with increasing the production of breast milk. Further, studies have shown that, by using CAM, postpartum mothers’ general well-being can be enhanced by reducing weight, relieving constipation and improving symptoms of insomnia [9–14]. Some of them also believe that naturopathy using abdominal hot stone application or massage post-delivery can improve uterine involution and abdominal muscle tone [15]. There are controversial or contradictory findings about the most common types of CAM used during post-partum period. In the states of United, the mind–body practices, followed by manipulative body therapies and biological-based therapies [11] were the commonest practice among postpartum women [10, 11] On the other hand, in Malaysia, naturopathy with abdominal message and herbs usage are the commonest practice in local studies [15, 16]. Studies have also shown that relaxation therapy, aromatherapy and massages have increased in popularity among postpartum women to alleviate their health problems, including general malaise, stress and postpartum depression [17].

## Methods

### Setting

This is a cross-sectional study of patients registered with a primary healthcare clinic in Keramat, Kuala Lumpur, Malaysia. It covers a total population of 66,605 people [18]. The study was conducted over a three-month period from January 2018 to March 2018.

### Inclusion criteria

The inclusion criteria for the study were women aged 18 years and above, who had their latest delivery within 12 months of the study period. The exclusion criteria included those who were intellectually challenged or clinically unstable during the study period. The sample size was calculated using the Lemeshow formula [19] based on the prevalence rates of CAM usage of 24.2 and 35.4%, according to different age groups in a previous study by Birdee GS et al. in the United States [10]. Sample size is calculated with the power of study 80% and significance level, α at 0.05 with 95% confidence interval, using two sample proportion formula as below:

$$ {\displaystyle \begin{array}{l}\mathrm{n}={\frac{\left[{\mathrm{Z}}_{1-\mathrm{a}2}\left(\surd 2\mathrm{P}\left(1-\mathrm{P}\right)\right)+{\mathrm{Z}}_{1-\beta}\left(\surd \mathrm{P}1\left(1-\mathrm{P}1\right)+\mathrm{P}0\left(1-\mathrm{P}0\right)\right)\right]}{{\left(\mathrm{P}1-\mathrm{P}0\right)}^2}}^2\times 2\\ {}\mathrm{Z}1\hbox{-} \upalpha /2=1.96\ \mathrm{for}\ \upalpha =0.05;\mathrm{z}1\hbox{-} \upbeta =0.84\ \mathrm{for}\ 80\%\mathrm{power}\ \mathrm{of}\ \mathrm{study}\\ {}\mathrm{P}1=\mathrm{Estimated}\ \mathrm{proportion}\ \mathrm{of}\ \mathrm{one}\ \mathrm{population}\ \mathrm{of}\ \mathrm{the}\ \mathrm{outcome}\\ {}\mathrm{P}0=\mathrm{Estimated}\ \mathrm{proportion}\ \mathrm{of}\ \mathrm{second}\ \mathrm{population}\ \mathrm{of}\ \mathrm{the}\ \mathrm{outcome}\\ {}\mathrm{P}=\frac{\left(\mathrm{P}1+\mathrm{P}0\right)}{2}\end{array}} $$

The significant and important factors obtained from the previous research (3) are calculated. The largest sample size is yielded from the age (years) factor. Therefore, the formula is calculated as below: Age 18–30 = P1, Age 30–49 = P0, P1 = 0.242, P0 = 0.354, P = (0.242 + 0.354)/2 = 0.298.

$$ {\displaystyle \begin{array}{l}\mathrm{n}={\frac{\left[1.96\left(\surd 2(0.298)\left(1-0.298\right)\right)+0.84\left(\surd 0.242\left(1-0.242\right)+0.354(0.646)\right)\right]}{{\left(0.242-0.354\right)}^2}}^2\times 2\\ {}=522\end{array}} $$

Account for 80% of respondent rate = 522/0.8 = 652.5; Account for 90% eligibility rate = 652.5/0.9 = 725. The final sample size calculated is 725, which takes into account a non-respondent rate of 20%, non-eligibility rate of 10, 80% power and a significance level of 0.05.

### Data collection

A face-to-face interview was conducted using an adapted, structured questionnaire. After obtaining ethical approval, we approached each participant and explained the nature of the study before obtaining written consent of their participation. A systematic sampling method was used to recruit respondents in this study. The estimated number of postpartum women who came for postnatal follow-up was 30 patients per day, or 1800 patients in 3 months. Since the estimated sample size was 725, a sampling interval of two was used – as a constant – during recruitment for the study. A starting number of 1 was selected randomly from the Maternal Child Health registration counter.

### Data collection instrument

The adapted questionnaire was structured based on the Maternal Health Record Book published by the Ministry of Health, Malaysia (MOH) [20]. The questionnaire was initially prepared in English by the author. The contents were reviewed by an expert panel comprising three family physicians. Forward and backward translations were performed by two certified translators into Malay and English. The questionnaire was a self-administered type of questionnaire, divided into two sections. The first section included patients’ sociodemographic and obstetrical information. The second section explored the experience, types, durations, the reasons for CAM use and the satisfaction level with CAM use. A pilot study involving 73 patients, 10% of the actual sample size, was conducted to pretest the questionnaire and estimate the likely response rate. Recruitment was done via the systematic-sampling method, with every one in two patients registered at the maternal and child-health clinic for postnatal clinical follow up at the same clinic. Findings from this pilot study were not included in the data analysis of the actual study.

### Operational definition

A postpartum mother is an individual in the period between having given birth to a child up to 1 year after delivery [21]. CAM use, in this study, is defined as the use of any of the following therapies: biological-based therapies, manipulative body therapy, mind–body therapy, whole medical systems – including acupuncture, Ayurveda and homeopathy, energy medicine, etc. [3].

### Data analysis

Statistical Package for Social Sciences (SPSS) version 23.0 was used to analyze the data collected in the study. Descriptive analysis was used to describe the characteristics of the respondents in terms of frequencies, percentages, means, and standard deviations. In this study, the chi-square test was used for categorical data and the independent t-test (or Mann–Whitney U test) for the continuous data to identify the relationships between the usage of CAM among postpartum mothers and socio-demographic and obstetrical factors. Multivariate logistic regression was used to identify the predictors of CAM usage. All variables with the *p*-value < 0.25 in the univariate analysis, as well as the clinically significant variables, were entered into the multiple logistic regression. The dependent variable was CAM use among postpartum mothers. The independent variables were age, ethnicity, religion, education level, employment status, household income, parity, delivery method and maternal complications.

### Ethical approval

Ethical approval was obtained from the Medical Research and Ethics Committee (MREC), Ministry of Health Malaysia (NMRR-16-2474-32,261) and MREC JKEUPM, Universiti Putra Malaysia (UPM/TNCPI/RMC/1.4.18.2) prior to data collection.

## Results

### Socio-demographic characteristics

A total of 735 postpartum women were recruited for this study, with a response rate of 98.6%. No missing data were noted in our study. Table 1 shows the socio-demographic and obstetrical information of the study respondents. The mean age of the study respondents was 31.2 ± 4.8, with a range of 18–34. The respondents were predominantly Malay Muslims (94.5%), with more than half of them having attained tertiary educational level (66.1%). Only 5.8% of the respondents reported complications following delivery.

**Table 1:** Sociodemographic and Obstetric data of study respondents in Klinik Keramat, Malaysia (*n* = 725)

Variable	Frequency N (%)	Mean ± SD/
–	–	Median (IQR)
Age, years		31.2 ± 4.8
Race		
Malay	673 (92.8)	
Chinese	26 (3.6)	
Indian	10 (1.4)	
Others	16 (2.2)	
Religion		
Muslim	685 (94.5)	
Buddhism	16 (2.2)	
Hindu	7 (1.0)	
Others	17 (2.3)	
Education Level		
No formal education	1 (0.1)	
Primary school	14 (1.9)	
Secondary school	231 (31.9)	
College/University	479 (66.1)	
Employment status		
Unemployed	266 (36.7)	
Employed	459 (63.3)	
Household Income per month (RM)		4000 (3000)
Parity		
Primiparous	276 (38.1)	
Multiparous	449 (61.9)	
Delivery method		
Spontaneous vaginal delivery	489 (67.4)	
Assisted vaginal delivery	21 (2.9)	
Caesarian section delivery	215 (29.7)	
Complication after Delivery		
No	683 (94.2)	
Yes	42 (5.8)	
Deep vein thrombosis	2 (0.3)	
Bleeding	13 (1.8)	
Perineal infection	1 (0.1)	
Depression	3 (0.4)	
Others^a^	23 (3.2)	
Duration of CAM use (days)		
1–29	200 (32.3)	30 (38)
30–44	236 (38.1)	
> 45	184 (29.7)	–

^a^Wound breakdown, retained placenta, haemorrhoids, neck pain, late onset pregnancy induced hypertension, anemia & breast engorgement

### Prevalence of CAM use

The prevalence of CAM use among postpartum mothers was 85.5% (*n* = 620/725). Most respondents used CAM for between 30 to 44 days, with a median of 30 days. The median monthly expenditure on CAM was 250 Malaysian Ringgits (USD = 60.35). Table 2 shows the different types of CAM utilized by postpartum mothers who attended the clinic. The most common type of CAM used was manipulative body therapy (84.1%), followed by biological-based therapy (33.1%). In our study, we found that neonatal jaundice was also found in 57.1% of newborns whose mothers consumed herbal medicine.

**Table 2:** Types of CAM used by postpartum mothers in Klinik Keramat, Malaysia (*n* = 725)

Types of CAM	Frequency (N)	Percentage (%)
Manipulative body therapy	610	84.1
Massage	555	76.6
Hot stone compression	488	67.3
Body wrapping	447	61.7
Reflexology	6	0.8
Biological-based therapies	240	33.1
Herbal Medicine	240	33.1
Mind body therapy	3	0.4
Yoga	2	0.3
Qi Gong	1	0.1
Whole medical system	12	1.7
Homeopathy	10	1.4
Acupuncture	1	0.1
Ayurveda	1	0.1
Energy Medicine	0	0
Sauna and cupping	6	0.8

### Reasons for CAM use and source of information

As shown in Table 3, more than half of the respondents opted to use CAM as they had observed positive results from other CAM users (52.1%). However, a quarter of them (27.5%) stated that they simply wanted to ‘try it out’. Most postpartum mothers who had used CAM reported improved overall physical health (61.8%) with a reduction in stress after delivery (15.2%), and an increased amount of breast milk (13.7%). Most users stated that they were satisfied with the CAM they had been using (80.3%).

**Table 3:** Reasons of CAM use and source of information among postpartum mother in Klinik Keramat, Malaysia (*n* = 725)

Variables	Frequency (N)	Percentage (%)
Reasons for CAM use		
Good example from other users of CAM	323	52.1
Just to try out	171	27.6
Routine practice and family tradition	73	11.8
To treat other diseases (co-morbidity)	27	4.4
Believe modern medicines are harmful	22	3.5
Not satisfied with conventional medicine	4	0.6
How CAM has helped		
Improved overall physical health	383	61.8
Reduced stress after delivery	94	15.2
Increased breast milk	85	13.7
Lose weight	43	6.9
Others^a^	15	2.4
Satisfaction level		
Very unsatisfied	0	0
Unsatisfied	4	0.6
Neutral	71	11.5
Satisfied	498	80.3
Very satisfied	47	7.6
Source of Information		
Family	527	85
Friends	45	7.3
Media	36	5.8
Doctor	7	1.1
Others^b^	5	0.8

^a^reduced body ache, achieved body relaxation, attained body heat, speed up wound healing and bleeding cessation & no effect at all

^a^Neighbours, know by themselves & village midwife

### Sources of information on CAM use

A majority of the users reported receiving information on CAM from their family members (85%), followed by friends (7.3%) and media (5.8%).

### Factors associated with CAM use

Table 4 shows the association between CAM use among postpartum mothers and socio-demographic factors and obstetrical factors. Unadjusted univariate logistic regression shows that ethnicity, religion, educational level and delivery method have a significant relationship with CAM use in postpartum mothers. Subsequently, multivariate logistic regression was used to analyze all the independent variables with *p*-values lower than 0.25 in the univariate logistic regression to determine the predictors of CAM use in postpartum mothers.

**Table 4:** Comparison in categorical variables between postpartum mothers in KK Keramat with or without CAM use in Malaysia (*n* = 725)

Variables	CAM users, *n* = 620(%)	Non-CAM users, *n* = 105(%)	*p*-value
Age	31.1 ± 4.7	32.0 ± 4.9	0.064
Monthly household income	4000 (3200)	4000 (2950)	0.138
Ethnicity			< 0.001
Non-Malay	22 (42.3)	30 (57.7)	
Malay	598 (88.9)	75 (11.1)	
Religion			
Non-Muslim	15 (37.5)	25 (62.5)	< 0.001
Muslim	605 (88.3)	80 (11.7)	
Education Level			< 0.001
Secondary level and below	194 (78.9)	52 (21.1)	
Tertiary level	426 (88.9)	53 (11.1)	
Employment status			0.102
Unemployed	220 (82.7)	46 (17.3)	
Employed	400 (87.1)	59 (12.9)	
Parity			0.275
Primiparous	231 (83.7)	45 (16.3)	
Multiparous	389 (86.6)	60 (13.4)	
Delivery method			< 0.001
Vaginal delivery	465 (91.2)	45 (8.8)	
Caesarean section	155 (72.1)	60 (27.9)	
Complication after delivery			0.164
No	581 (85.1)	102 (14.9)	
Yes	39 (92.9)	3 (7.1)	–

Table 5 shows that Malay postpartum mothers are 4.414 times (95% confidence interval [CI]: 1.176–16.562, *p* = 0.028) more likely to use CAM than non-Malays. Further, Muslims are also at 5.01 times more likely to use CAM than non-Muslims (95% CI: 1.136–22.082, *p* = 0.033). Postpartum mothers who attained a tertiary educational level are also 2.561 times more likely to use CAM than those who only have an education background of secondary level and below (95% CI: 1.587–4.133, *p* < 0.001). Finally, mothers who delivered via vaginal delivery had a higher likelihood of using CAM in comparison to those who delivered through caesarean section (odds ratio [OR]: 5.660, 95% CI: 3.454–9.276).

**Table 5:** Multiple Logistic Regression of the associated factors of CAM use among postpartum women in Klinik Keramat, Malaysia (*n* = 725)

Variables	Adjusted OR	95% CI (Lower, upper)	*P* value
Delivery Method, Vaginal delivery	5.66	3.454 9.276	< 0.001
Caesarean section	1		
Religion, Muslim	5.01	1.136 22.082	0.033
Non-Muslim	1		
Ethnicity, Malay	4.414	1.176 16.562	0.028
Non-Malay	1		
Education level, Tertiary level	2.561	1.587 4.133	< 0.001
Secondary level and below	1		
Employment status, employed	1.418	0.932 2.156	0.103
Unemployed	1		
Complication after delivery, Yes	3.023	0.796 11.480	0.104
No	1		
Age	0.959	0.914 1.007	0.093
Monthly Household income	1	1.000 1.000	0.163

*OR* Odds ratio, *CI* Confidence Interval

## Discussion

The prevalence of CAM usage among postpartum mothers in the study population was high (85.5%), which is consistent with previous local studies and other Asian countries, which have shown prevalence values between 45 and 95.4% [9, 13–16, 22, 23]. The prevalence of CAM use reported in a tertiary hospital in Malaysia was 87.3 and 83.2% in a district health clinic in Malaysia [15, 16]. A possible reason for the high consumption of CAM among postpartum mothers in Asia could be their belief that CAM can help mothers recover strength, relax, minimize the risk of infection, restore their body-heat imbalance and facilitate uterine recovery [24]. Furthermore, most CAM users think that CAM is safe to use, along with being easily available and cheap [15, 25, 26]. Our study also shows that the median expenditure on CAM per month by the postpartum mothers was only RM250 (USD = $60.35). Further, the median duration of CAM use among participants was approximately 30 days (IQR: 38). This corresponds to the duration of the confinement period in most Asian countries, which is approximately 30–40 days [24].

The most common CAM modality used in this study is manipulative body therapy, which includes massages, reflexology, hot stone compression and body wrapping. The finding is similar with another two local studies [15, 16]. Next is biological-based therapy, which comprises mainly herbal medicine and whole medical systems including acupuncture, Ayurveda and homeopathy. Our findings are consistent with a study in China, where Chinese mothers in the postpartum period predominantly utilized manipulative therapy, most often opting for acupressure and massage, followed by herbal medicine [14].

Our study showed that herbal medicine consumption among postpartum mothers was the second-most common modality of CAM usage. Surprisingly, among these mothers who consumed herbal medicine, more than half of them (57.1%) reported neonatal jaundice in their newborn. The incidence rate was higher than that found in another local study conducted in a teaching hospital; a 49.1% rate of neonatal jaundice was reported among postpartum mothers who took herbal medicine after delivery [13].

In this study, we also determined that over half of the mothers had opted to use CAM therapies because they had seen positive results from other CAM users (52.1%). This was followed by individuals who simply wanted ‘to try out’ those CAM therapies (27.6%). We observed that the majority of our respondents had been influenced by their family members (85.0%), reporting that they were their sole source of information regarding CAM, while only 1.1% received information regarding CAM from medical practitioners. Again, these findings were not surprising as prior studies on CAM use among postpartum women have also indicated that a majority of mothers in the postnatal period have used CAM on the recommendation of their family members, primarily their mothers or mothers-in-law, while very few of them had discussed this information with doctors [11, 13, 15, 23, 27–30]. The above results were also consistent with those from the literature, which suggested that CAM utilization had been constantly passed from one generation to another. The positive effects reported by one’s ancestors, along with the respect given to tradition, has ensured that CAM is still widely utilized [30, 31].

Studies have stated that the utilization of CAM is predominantly based on a patient’s perception, and the perceived natural features of CAM were responsible for their therapeutic effects [32]. This is similar to our result, in that most mothers in our sample population were satisfied with the use of CAM (80.3%) and believed that it improved their overall physical health (61.8%). Others have claimed that CAM reduces psychological stress post-delivery (15.2%) and increases breast milk production (13.7%). Only 6.9% of the participants observed weight loss while practising CAM.

Our study has demonstrated that Malay postpartum mothers have a higher likelihood of using CAM. The study, which was carried out in a tertiary hospital in Malaysia, has demonstrated similar findings, whereby Malay mothers ingest significantly more herbs in the postnatal period [13]. Chinese was reported to be less likely to consume CAM in another local study [15]. An explanation for this could be that herb ingestion is deeply rotten in traditional Malay beliefs [33].

Our study also showed that Muslim mothers had a higher probability of using CAM in the postnatal period in comparison to non-Muslims mothers. The significant association between Islamic beliefs and CAM use can perhaps be explained by the fact that the utilization of CAM has become a part of Islamic faith and that the cultural traditions have been greatly integrated into Islam [34, 35]. In addition, the fact that the population in this study were primarily Malay and Muslim may indirectly explain why being Muslim is one of the predictors of CAM use.

Similar to other previous studies, our study has reported that there is a higher tendency of postpartum mothers with tertiary education to utilize CAM [10, 11, 15, 22]. This could be due to the fact that women with tertiary education have higher chances of being employed and consequently have higher earnings and can afford CAM. Furthermore, those with a higher education level may have a broader knowledge of CAM. This will further increase their self-empowerment in making their own health decisions [36].

It has been demonstrated in this study that postpartum women who had vaginal delivery use CAM more than those with other methods of delivery. These results are similar to those of a study conducted in Taiwan, which reported that those who had a vaginal delivery are more likely to use CAM compared to those who delivered via caesarean section [22]. Studies have shown that manipulative body-based practices are the most commonly practised method of CAM among postpartum mothers. This explains why those who delivered via caesarean section did not prefer to use CAM, because this therapy requires bodily movements that might be difficult and painful for mothers post caesarian section [10, 11, 14, 22, 37].

Our study reported no association between age and CAM use among postpartum mothers, which is corroborated by other studies [9, 13, 22]. There are conflicting results for the relationship between CAM use among postpartum mothers and monthly household income [10, 27, 28]. However, our study found no significant relationship between monthly household income and CAM use in postpartum mothers [13].

In terms of obstetrical factors, this study has shown that complications after delivery and maternal parity are not associated with CAM use. To date, to the best of our knowledge, there has been no investigation into the association between CAM practice and the presence of postpartum complications. This study could serve as the first to explore the association between postpartum complications and CAM use. Additionally, our study has shown no association between maternal parity and CAM use. Although most studies previously have reported comparable findings [14, 22, 27], a local study done by Teoh et al. has shown that multiparous mothers have used significantly more CAM than primiparous mothers [13]. Further investigation would be required to better examine any associations between these factors and CAM usage.

### Strengths and limitations

The sample size of this study is relatively larger than those in other existing literature. In addition, this study has not only identified socio-demographic factors affecting CAM use, but also explored the obstetrical factors that affect it, which has not been investigated by previous local studies, especially in the primary healthcare setting. Furthermore, we have also looked into additional data on experiences and perspectives towards CAM use in the postpartum period. One distinct finding in this study is the high incidence of neonatal jaundice (57.1%) among mothers who took herbal medicines during the postpartum period. However, future research should be done to investigate the impact of CAM on this issue.

This study has some limitations. Firstly, time constraints were one of the most important factors that limited us from recruiting participants from other centres. Therefore, future studies should consider multiple centres. Secondly, since this is a cross-sectional study, data on every respondent were collected only once within the specified period. Therefore, we were unable to identify any temporal associations between the socio-demographic and obstetrical factors and CAM use. Therefore, only an association and no causal relation can be inferred from this study.

## Conclusion

It was found that the prevalence of CAM use was high (8 out of 10) among postpartum mothers. Postpartum mothers who are Malay, Muslim, have a higher educational level and who have had spontaneous vaginal delivery tend to use CAM more. Manipulative body therapies, including massage, reflexology, hot stone compression and body wrapping, were the most widely used CAM modalities, followed by biological-based therapies. However, our study showed that more than half of the mothers who consumed herbal medicine reported neonatal jaundice in their newborn. Urgent intervention, such as education regarding the risks of herbal consumption and neonatal jaundice during the antenatal period is required.

## Data Availability

The data is available and will be provided upon request to the corresponding author.

## Abbreviation

CAM: Complementary Alternative Medicine
SPSS: Statistical package for social sciences;
WHO: World Health Organization
MREC: Medical Research Ethical Committee
MOH: Ministry of Health
USD: United States Dollar
RM: Ringgit Malaysia
OR: Odds Ratio
CI: Confidence Interval
IQR: Interquartile Range
